# Epilepsy surveillance in normocephalic children with and without prenatal Zika virus exposure

**DOI:** 10.1371/journal.pntd.0008874

**Published:** 2020-11-30

**Authors:** Karen Blackmon, Randall Waechter, Barbara Landon, Trevor Noël, Calum Macpherson, Tyhiesia Donald, Nikita Cudjoe, Roberta Evans, Kemi S. Burgen, Piumi Jayatilake, Vivian Oyegunle, Otto Pedraza, Samah Abdel Baki, Thomas Thesen, Dennis Dlugos, Geetha Chari, Archana A. Patel, Elysse N. Grossi-Soyster, Amy R. Krystosik, A. Desiree LaBeaud

**Affiliations:** 1 Mayo Clinic, Department of Psychiatry and Psychology, Jacksonville, Florida, United States of America; 2 Windward Islands Research and Education Foundation, St George’s University, St George’s, Grenada, West Indies; 3 St George’s University School of Medicine, Department of Physiology, Neuroscience, and Behavioral Sciences, St. George’s, Grenada, West Indies; 4 Ministry of Health, Government of Grenada, West Indies; 5 Biosignal Group Inc., Boston, Massachusetts, United States of America; 6 New York University School of Medicine, Department of Neurology, New York, New York, United States of America; 7 Department of Biomedical Sciences, University of Houston College of Medicine, USA; 8 Children’s Hospital of Philadelphia, Philadelphia, Pennsylvania, United States of America; 9 SUNY Downstate Health Sciences University, Brooklyn, New York, United States of America; 10 Boston Children’s Hospital, Department of Neurology, Boston, Massachusetts, United States of America; 11 Stanford University School of Medicine, Department of Pediatrics, Stanford, California, United States of America; University of Costa Rica, COSTA RICA

## Abstract

Children with Congenital Zika Syndrome and microcephaly are at high risk for epilepsy; however, the risk is unclear in normocephalic children with prenatal Zika virus (ZIKV) exposure [Exposed Children (EC)]. In this prospective cohort study, we performed epilepsy screening in normocephalic EC alongside a parallel group of normocephalic unexposed children [Unexposed Children (UC)]. We compared the incidence rate of epilepsy among EC and UC at one year of life to global incidence rates. Pregnant women were recruited from public health centers during the ZIKV outbreak in Grenada, West Indies and assessed for prior ZIKV infection using a plasmonic-gold platform that measures IgG antibodies in serum. Normocephalic children born to mothers with positive ZIKV results during pregnancy were classified as EC and those born to mothers with negative ZIKV results during and after pregnancy were classified as UC. Epilepsy screening procedures included a pediatric epilepsy screening questionnaire and video electroencephalography (vEEG). vEEG was collected using a multi-channel microEEG® system for a minimum of 20 minutes along with video recording of participant behavior time-locked to the EEG. vEEGs were interpreted independently by two pediatric epileptologists, who were blinded to ZIKV status, via telemedicine platform. Positive screening cases were referred to a local pediatrician for an epilepsy diagnostic evaluation. Epilepsy screens were positive in 2/71 EC (IR: 0.028; 95% CI: 0.003–0.098) and 0/71 UC. In both epilepsy-positive cases, questionnaire responses and interictal vEEGs were consistent with focal, rather than generalized, seizures. Both children met criteria for a clinical diagnosis of epilepsy and good seizure control was achieved with carbamazepine. Our results indicate that epilepsy rates are modestly elevated in EC. Given our small sample size, results should be considered preliminary. They support the use of epilepsy screening procedures in larger epidemiological studies of children with congenital ZIKV exposure, even in the absence of microcephaly, and provide guidance for conducting epilepsy surveillance in resource limited settings.

## Author summary

Epilepsy is a clinical concern in children with Congenital Zika Syndrome and microcephaly; however, it is unknown whether epilepsy rates are elevated in normocephalic children with prenatal Zika virus exposure. We found a modest elevation of epilepsy rates in the first year of life of exposed, relative to unexposed, children, despite no other neurological manifestations. Seizures in exposed children had features of focal, rather than generalized, epilepsy and were well controlled with carbamazepine. Our findings suggest that epilepsy surveillance should be included in the neurodevelopmental assessment of children with prenatal Zika virus exposure, even in the absence of neurologic manifestations at birth.

## Introduction

Epilepsy is a clinical concern in children with Congenital Zika Syndrome (CZS). Epilepsy is diagnosed in 48–96% of children with CZS when microcephaly is present [1–5]. It is unclear whether the risk for epilepsy is elevated in normocephalic children with prenatal Zika virus (ZIKV) exposure [Exposed Children (EC)]. We aimed to address this question by conducting epilepsy surveillance in a prospective cohort of normocephalic EC alongside a parallel group of normocephalic unexposed children (UC).

The teratogenicity of ZIKV is well established [6]; ZIKV is able to cross the placental barrier, disseminate to the fetus, and target cortical progenitor [7] and glial cells [8], leading to malformations of cortical development (MCD). MCDs can be severe and visually obvious, such as microcephaly [9], but also focal and radiographically occult [10]. In animals and humans, ZIKV-induced brain malformations are found in the absence of microcephaly [10–12] and are not reliably detected with standard antenatal diagnostic tools such as ultrasound [10]. This makes it difficult to discern whether EC were protected from ZIKV neurotropism during gestation or whether they harbor subtle pathology that might become epileptogenic at later stages of development.

In 2016, the tri-island nation of Grenada, Carriacou, and Petit Martinique experienced a ZIKV outbreak with peak transmission from May through October of 2016 [13]. In April 2016, the Ministry of Health and the Windward Islands Education and Research Foundation (WINDREF) began recruiting pregnant women for participation in a ZIKV surveillance study during their antenatal and postnatal care appointments at local health centers, regardless of whether they presented with ZIKV symptoms. Outcome assessments were performed on their offspring between 1 and 12 months of age and again between 12 to 30 months of age to determine the spectrum of neurodevelopmental sequelae associated with prenatal ZIKV exposure. We administered an epilepsy surveillance protocol in the context of this larger, multidisciplinary effort.

Epilepsy surveillance in low- and middle-income countries (LMIC) is often limited by the absence of epilepsy specialists and diagnostic tools [14], although LMICs bear a disproportionate burden of epilepsy [15,16]. Insufficient surveillance can increase the time between epilepsy onset and treatment [17] and increased time to treatment can compound the negative effects of epilepsy [18]. A well-designed pediatric epilepsy screening questionnaire can improve epilepsy detection in large populations of children and in the absence of specialist care [19,20]. We utilized a pediatric epilepsy screening questionnaire that was designed to be administered by non-specialist health care providers in resource-limited settings and validated to discriminate focal from generalized seizures [20]. In a subset of children, we also obtained video electroencephalography (vEEG), which was independently interpreted remotely by at least two expert pediatric epileptologists, who were blinded to the child’s ZIKV exposure status. This approach allowed us to bridge the gap between remote epilepsy specialists and the local pediatrician (TD) who performed diagnostic evaluations and provided clinical care for the children in our study. The aim of our study was to detect and characterize epilepsy in EC and to compare epilepsy incidence rates among EC and UC to global incidence rates at one year of life.

## Methods

### Ethics statement

Institutional Review Board approval was obtained at St George’s University (IRB#16061) and Stanford University (IRB#45242). Written informed consent was obtained from all mothers who participated in this study. There was no financial compensation.

### Participants

**Fig 1:**
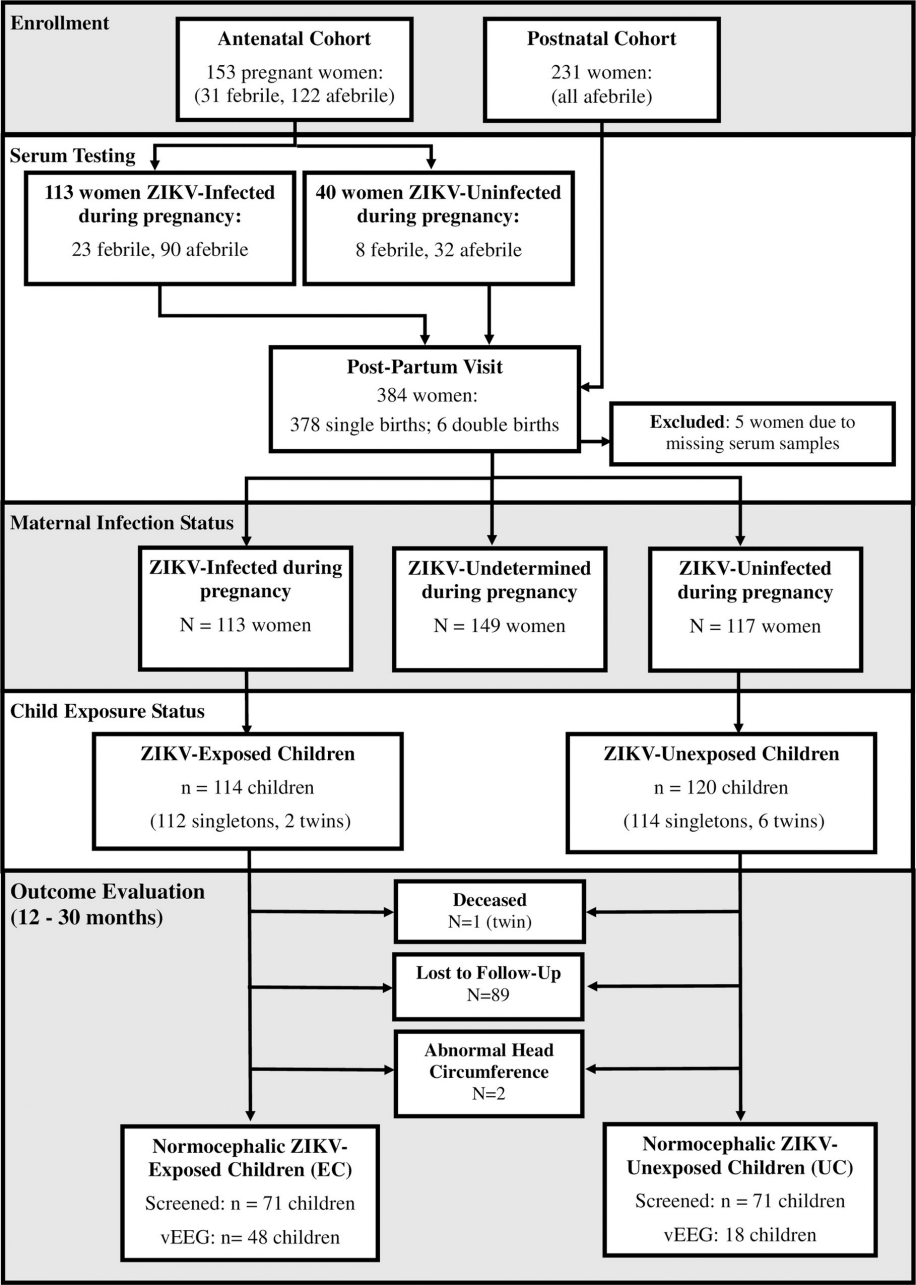
Flow diagram of enrollment, serum testing, and outcome evaluation.

Mother-infant pairs were recruited between April 2016 and March 2017 from ten public health centers across all parishes of Grenada. A total of 384 mothers signed informed consent to participate (Fig 1). Mothers were recruited during the antenatal or postnatal period:

*Antenatal Cohort*: A total of 153 pregnant women were recruited during pregnancy. In this cohort, maternal serum was collected at a single time point during the prenatal period and a follow-up time point during the postnatal period (0–12 months postpartum).

*Postnatal Cohort*: A total of 231 women were recruited during the postnatal period. Maternal serum was collected at a single time point during the postnatal period (0–12 months postpartum).

### Laboratory testing

Maternal serum samples were initially assessed for flavivirus exposure with indirect IgG capture Enzyme-Linked Immunosorbent Assay (ELISA) using dengue virus (DENV_1-4_) antigen [21] andwere further assessed using a multiplexed assay on a nanostructured plasmonic gold (pGOLD) platform (Nirmidas Biotech, Palo Alto, CA) for the detection of IgG and IgG avidity against ZIKV and DENV antigens [22]. The pGOLD IgG immunoassay was used to cross-validate ELISA results and distinguish ZIKV from DENV antigens, as it has demonstrated sensitivity and specificity to ZIKV greater than 90% and 98%, respectively, in the convalescent phase [22]. IgG avidity testing provided further data on the timing of ZIKV and DENV infection [22]. In addition, reverse transcription polymerase chain reaction (RT-PCR) was used in seven mothers who were febrile at time of assessment [23]. Mothers were provided feedback on their ZIKV status by the Grenada Ministry of Health. Research staff administering follow-up assessments remained blinded to maternal ZIKV status.

### Head circumference classification

Infants’ occipitofrontal head circumference was measured at birth and at a follow-up visit between 12 and 30 months of age. Measurements were made by 3 independent raters and the mean of these measurements was utilized. Normocephalic was defined as occipitofrontal head circumference between the 3^rd^ and 97^th^ percentile for sex and age in accordance with World Health Organization Child Growth Standards [24].

### Inclusion criteria

*EC Group*: Children were included in the EC group if: (1) they were born to mothers classified as “ZIKV-Infected” during pregnancy based on positive prenatal laboratory results (ELISA and pGOLD) for ZIKV, with avidity testing showing infection in the past 6 months [22]; and (2) they were normocephalic at birth and at the follow-up visit; and (3) they completed the epilepsy screening questionnaire. Given that inclusion in the EC group is based on positive *prenatal* maternal laboratory results for ZIKV, this excluded all children born to mothers in the postnatal cohort (i.e, maternal prenatal serum was not collected in this group).

*UC Group*: Children were included in the UC group if: (1) they were born to mothers who were classified as “ZIKV-Uninfected” during pregnancy, based on negative laboratory results for ZIKV on both the ELISA and pGOLD assays; and (2) they were normocephalic at birth and at the follow-up visit; and (3) they completed the epilepsy screening questionnaire. Maternal classification as “ZIKV-Uninfected” required consistently negative laboratory results for ZIKV on both the prenatal and postnatal serum samples in the antenatal cohort and the postnatal sample alone in the postnatal cohort.

### Epilepsy surveillance protocol

An epilepsy surveillance protocol was administered to EC and UC during a follow-up evaluation when they were between 12 and 30 months of age (Fig 1). Our epilepsy surveillance protocol consisted of a pediatric epilepsy screening questionnaire [20], as well as vEEG in a subset of children whose caregivers consented to this procedure. We utilized a questionnaire that has successfully differentiated focal and generalized epilepsy in Zambian and Tanzanian cohorts [20]. One question was added to probe for infantile spasms. The questionnaire was reviewed for cultural appropriateness by a focus group of Grenadian women and suggested adaptations were implemented. It was administered to the primary guardian at the follow-up evaluation by local research staff with undergraduate or graduate degrees in Psychology who were trained in seizure safety, detection, and classification through online content made available through the Epilepsy Foundation. All research assistants were fluent in the local dialect and cultural idioms for describing seizures. Positive screenings were queried further to determine age of seizure onset and whether there was a family history of epilepsy and were referred for follow-up clinical evaluation with a local pediatrician.

The *micro*EEG® (www.biosignalgroup.com) monitoring system was used to collect vEEG because it has been validated in newborns [25] and was designed for use in field applications and high-noise environments [26–28]. The 26-channel system digitizes analogue EEG signals collected by electrodes, amplifies them, and using Bluetooth® wireless connectivity, transfers the physiological data to a dedicated laptop computer. The electrode caps come in different sizes and are made of elastic, weblike, fabric with electrodes attached in a configuration that conforms to the International 10/20 System. Research assistants were trained to apply the cap through instructional videos. After parting the child’s hair under each electrode, a small amount of electro-gel was applied through a hole in the electrode center to minimize the electrode-scalp impedance. We recorded for a minimum of 20 minutes along with a video recording of participant behavior time-locked to the EEG. vEEG data were transferred to a HIPAA compliant server and were independently reviewed by at least two board-certified pediatric epileptologists (DD, GC, AP), who were blinded to the child’s ZIKV status. The only accompanying information was date of birth, gender, and medications at time of recording. EEG recordings were interpreted as normal or abnormal using a standardized form. This form included category-specific designations for normal and abnormal findings. If abnormal, the following specifiers were included: seizure (focal, generalized, both); interictal epileptiform activity (focal, generalized, both, or multifocal); status epilepticus (focal or generalized); slowing (focal, diffuse, both); triphasic waves; burst suppression; and sleep (normal, abnormal-asynchronous sleep architecture, and abnormal-other). The standardized form also included specifiers for whether a recording was technically limited due to artifacts (but interpretable) or uninterpretable. At the end of data collection, a live group consensus meeting was held to provide all available clinical information to the epileptologists and resolve discordant EEGs.

### Epilepsy classification

Epilepsy classification followed International League Against Epilepsy (ILAE) guidelines for epilepsy diagnosis [29,30]. Epilepsy diagnosis was established by a local pediatrician (TD) after a full clinical evaluation in addition to consideration of data from our pediatric epilepsy screening questionnaire, vEEG interpretation forms, and information from the consensus meeting discussions.

### Statistical analysis

To address the primary study question, we compared the proportion of epilepsy cases in EC and UC to global incidence rates at one year of age [31–37] using a Bayesian probabilistic approach, given that epilepsy onset in all positive cases was in the first year of life. Fleiss’ Multirater Kappa coefficient was used as a measure of inter-rater reliability for absolute agreement of EEGs as normal versus abnormal on blinded review and was calculated prior to the group consensus meeting.

## Results

### Sample characteristics

Maternal ELISA and pGOLD results were concordant for flavivirus antigens; all of the samples that were positive for anti-flavivirus IgG by ELISA were also positive for anti-DENV, anti-ZIKV, or both using pGOLD, indicating that the pGOLD assay did not result in any false negatives. In addition, all samples that were negative for anti-flavivirus IgG by ELISA were also negative by pGOLD, indicating that the pGOLD assay did not result in any false positives.

Of the 384 women that signed consent to participate, a total of 113 were classified as “ZIKV-Infected” during pregnancy based on positive prenatal laboratory results for ZIKV and avidity testing showing infection within the past 6 months (Fig 1). Within this group, 96% also tested positive for DENV; however, avidity testing showed that DENV infection occurred more than 6 months prior to prenatal serum collection in 80%, indicating more remote infection. A total of 117 women were classified as “ZIKV-Uninfected” during pregnancy based on negative laboratory results on prenatal and postnatal serum (antenatal cohort) or postnatal serum alone (postnatal cohort). ZIKV status during pregnancy could not be determined for 149 women because (1) they had positive laboratory results for ZIKV on postnatal serum and there was no prenatal serum available (N = 144); or (2) they had negative ZIKV results on prenatal serology but positive ZIKV results on postnatal serology (N = 5).

A total of 114 children (112 singletons, 2 twins) were born to mothers classified as “ZIKV-Infected” during pregnancy. One of these children passed away in infancy (a twin); another child was found to have microcephaly at birth; and 41 were lost to follow-up because their caregivers declined to return for a follow-up outcome assessment or they could not be reached by phone. This resulted in 71 children that met criteria for the EC group. A total of 120 children (114 singletons, 6 twins) were born to mothers who were classified as “ZIKV-Uninfected” during pregnancy. One of these children was found to have macrocephaly at birth and 48 were lost to follow-up because their caregivers declined to return for a follow-up outcome assessment or they could not be reached by phone. This resulted in a total of 71 children in the UC group. The cohort of 71 EC and 71 UC represented 62% of the original cohort of EC (71/114) and 59% of the original cohort of UC (71/120). We observed no differences in household socio-demographics between mothers who consented for epilepsy screening and those who were lost to follow-up (Table 1).

**Table 1:** Socio-demographic characteristics of families lost to follow-up versus followed.

	Lost to Follow-Up (n = 89)	Followed (n = 142)	Chi-square	p-value
**Household Income (monthly Eastern Caribbean Dollars)**			3.42	0.49
Under 1,000	7 (8%)	11 (8%)		
1,001–2,000	17 (19%)	18 (13%)		
2,001–3,000	15 (17%)	29 (20%)		
Over 3,000	8 (9%)	21 (15%)		
Unknown/Refused	42 (47%)	63 (44%)		
**Maternal Education**			1.07	0.90
Primary	10 (11%)	20 (14%)		
Secondary	48 (54%)	77 (54%)		
College Degree	8 (9%)	9 (6%)		
Graduate Degree	4 (5%)	8 (6%)		
Unknown/Refused	19 (21%)	28 (20%)		

### Socio-demographic, clinical, and anthropometric comparisons

We observed no differences between EC and UC in household socio-demographics, sex distribution, rates of premature births, or neonatal complication rates (Table 2). Neonatal complications included respiratory distress (4 EC, 3 UC), jaundice (9 EC, 9 UC), and infection (1 EC, 2 UC). None of the EC had craniofacial disproportions, arthrogryposis, or motor abnormalities, which indicates that, in addition to being normocephalic, there were no other apparent neurological manifestations at birth. In addition, we observed no group differences between EC and UC in head circumference at birth, or age, head circumference, height, and weight at time of epilepsy surveillance (Table 2).

**Table 2:** Socio-demographic comparisons between exposed and unexposed children.

	**EC (N = 71)**	**UC (N = 71)**	**Chi-square**	**p-value**
**Child’s Sex**			0.72	0.40
Males	39 (55%)	44 (62%)		
Females	32 (45%)	27 (38%)		
**Household Income (monthly; Eastern Caribbean Dollars)**			1.74	0.78
Under 1,000	6 (8%)	5 (7%)		
1,001–2,000	7 (10%)	11 (15%)		
2,001–3,000	13 (18%)	16 (23%)		
Over 3,000	11 (16%)	10 (14%)		
Unknown/Refused	34 (48%)	29 (41%)		
**Maternal Education**			2.64	0.62
Primary	11 (15%)	11 (15%)		
Secondary	36 (51%)	36 (51%)		
College Degree	4 (9%)	4 (6%)		
Graduate Degree	6 (1%)	6 (8%)		
Unknown/Refused	14 (20%)	14 (20%)		
**Food Security**			2.80	0.42
Food Secure	23 (32%)	22 (31%)		
Food Insecure (Moderate)	15 (21%)	23 (32%)		
Food Insecure (Severe)	7 (10%)	7 (10%)		
Unknown/Refused	26 (37%)	19 (27%)		
**Prematurity**				
> 37 weeks gestation	54 (76%)	57 (80%)	1.67	0.43
≤ 37 weeks gestation	4 (6%)	6 (9%)		
Unknown/Refused	13 (18%)	8 (11%)		
**Neonatal Complications**				
No complication	52 (73%)	56 (79%)	2.82	0.25
Neonatal complications	14 (20%)	14 (20%)		
Unknown/Refused	5 (7%)	1 (1%)		
	**Mean (SD)**	**Mean (SD)**	**t-value**	**p-value**
**Mother’s age (years) at delivery**	28.68 (6.15)	28.29 (7.04)	-0.34	0.73
**Child’s age (months) at time of epilepsy screen**	21.77 (4.08)	22.11 (4.44)	0.47	0.64
**Child’s head circumference (cm) at birth**	33.43 (1.34)	33.21 (1.77)	-0.73	0.47
Child’s head circumference (*z-score) at time of epilepsy screen	0.45 (0.89)	0.53 (0.91)	0.46	0.65
Child’s weight (*z-score) at time of epilepsy screen	0.36 (1.12)	0.31 (1.19)	-0.24	0.81
Child’s height (*z-score) at time of epilepsy screen	0.13 (1.02)	-0.04 (0.99)	-0.88	0.38

EC = Normocephalic ZIKV-Exposed Children; UC = Unexposed Children

*z-scores calculated using World Health Organization Child Growth Standards

### Pediatric epilepsy screening questionnaire

Epilepsy screening questionnaires were administered to the caregivers of 71 EC and 71 UC. Two of 71 cases from the EC group screened positive versus 0/71 cases from the UC group. In both positive EC cases, the child’s mother indicated a history of multiple seizures, not associated with a fever, that were accompanied by loss of awareness and post-ictal fatigue. There was no indication of features consistent with infantile spasms or developmental regression. In both cases, the mother indicated features consistent with focal seizures (e.g., shaking/twitching on one side, eye/head-turning to one side). In both cases, seizure onset was in the first year of life, there was no family history of epilepsy, and the mothers had positive laboratory results for ZIKV during pregnancy but were asymptomatic. These two children were referred to a local pediatrician (TD) for an epilepsy diagnostic evaluation.

### Video electroencephalography

A total of 66 mothers signed consent for vEEG procedures (48 EC; 18 EC). vEEGs were interpretable in 62/66 cases (94%). Recordings were predominantly acquired while the child was awake (74%); however, 26% of the children fell asleep during the recording. The quality of the vEEGs was good in 49/66 cases (74%), technically limited but interpretable in 13/66 cases (20%), and uninterpretable in 4/66 cases (6%). Inter-rater reliability on initial blinded review was moderate (Fleiss’ Multirater Kappa: 0.47, 95% CI = 0.46–0.48) but comparable to other studies that used blinded review of EEGs [38]. Within the UC group, one vEEG was read as abnormal by two reviewers; both reviewers identified focal sharp waves in the centrotemporal region. Within the EC group, there were four vEEGs that were initially read as abnormal by one reviewer and normal by a second reviewer. Abnormal findings consisted of focal slowing in two cases and interictal focal epileptiform activity in two cases (one with epilepsy). For these four discordant cases, consensus was achieved during live unblinded joint review, with agreement that artifact could not be ruled out.

### Epilepsy cases

The two cases that screened positive for epilepsy had a follow-up evaluation by a local pediatrician. In the first case, epilepsy had already been clinically diagnosed at two weeks of age based on a history of at least two unprovoked seizures more than 24 hours a part. There was no family history of epilepsy or birth complications. An MRI obtained at 10 months of age was unremarkable. Seizures were initially well controlled with carbamazepine; however, there was a cluster of breakthrough seizures at 24 months. Two vEEGs at 11 and 12 months of age were normal. The second case was newly diagnosed with epilepsy based on a history of at least two unprovoked seizures more than 24 hours a part. Seizure onset was at three months of age, based on maternal report. Carbamazepine was started with good seizure control documented at 24-month follow-up. There was no family history of epilepsy or birth complications. Neuroimaging was not available. vEEG at 17 months of age was read as abnormal by one reviewer on initial blinded review, with findings of rare focal epileptiform discharges in the frontocentral region; however, consensus review determined that artifact could not be definitively ruled out.

### Epilepsy rates

**Fig 2:**
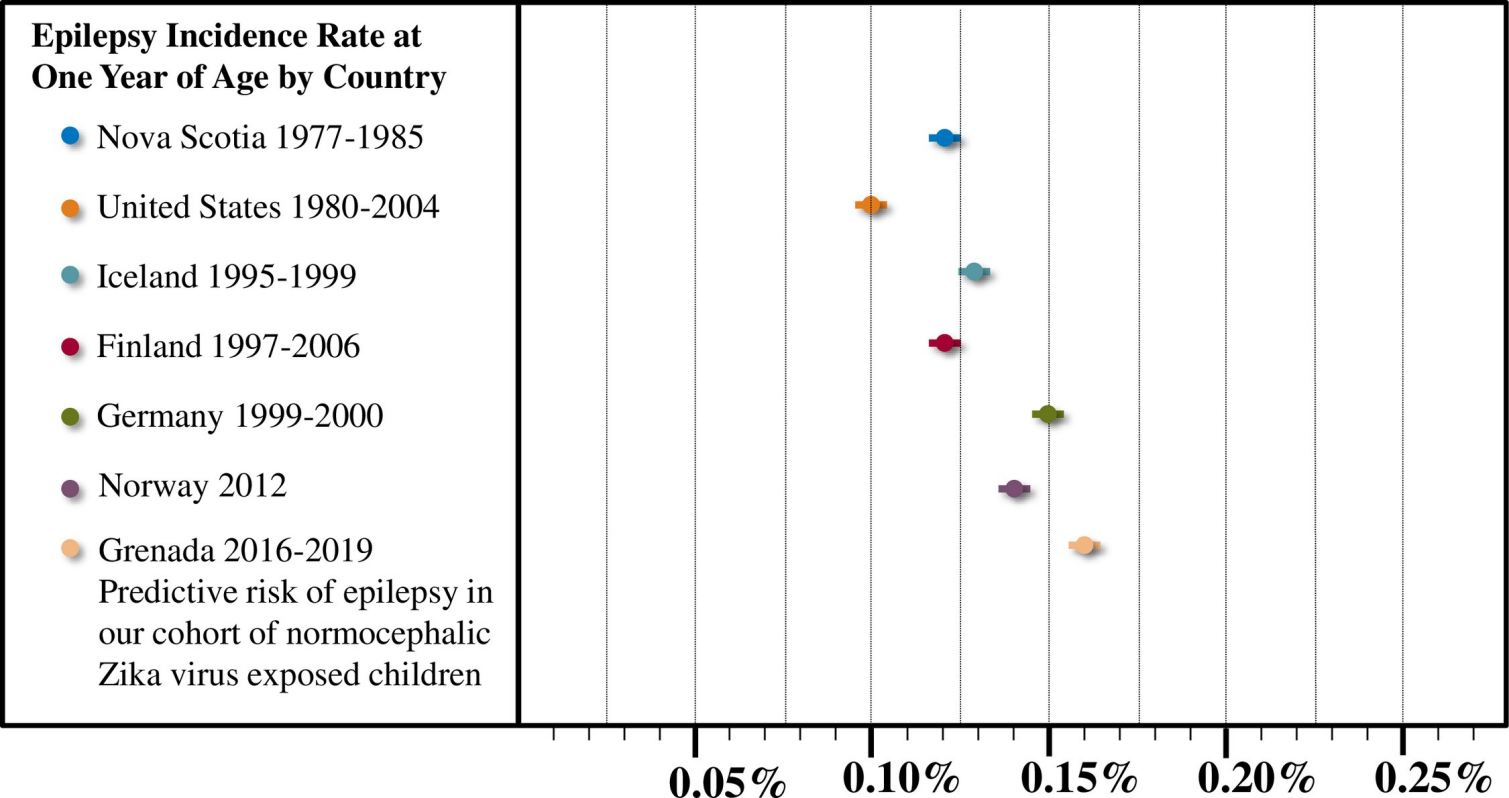
Global incidence rates of epilepsy at one year of life. Epilepsy incidence rates at one year of age range from 0.10% to 0.15% [Nova Scotia: 0.12%^31^; United States (Minnesota): 0.10%^33^; Iceland: 0.13%^35^; Finland (Helsinki): 0.12%^34^; Germany: 0.15%^37^; Norway: 0.14%^36^]. Bars represent 95% confidence interval (https://www.sample-size.net/confidence-interval-proportion). Using a Bayesian probabilistic approach, the predicted risk of epilepsy at one year of life in EC is 0.16%, which is higher than global incidence rates.

Epilepsy was observed in 2/71 EC (IR: 2.8%; 95% CI: 0.34–9.81%) and 0/71 UC (IR: 0.00; 95% CI: 0.00–5.36%). Global epilepsy incidence rates range from 0.10% to 0.15% in the first year of life [31–37] (Fig 2). Using a Bayesian probabilistic approach, assuming a uniform prior probability of epilepsy of 0.000 to 0.002, a binomial likelihood of a positive diagnosis, and a prior relative risk ratio of 1.00, the predicted risk of epilepsy in EC is 0.16%, which is greater than what would be predicted by global incidence rates (Fig 2). The predicted risk of epilepsy in UC is 0.1%, which is within the range of global incidence rates. The posterior relative risk ratio in EC (0.0016/0.001) of 1.6 is a small effect, indicating that the risk of epilepsy in the first year of life is elevated in EC but still considered a rare condition.

## Discussion

We performed epilepsy surveillance in a cohort of normocephalic children with prenatal ZIKV exposure (EC) alongside a parallel group of socio-demographically similar unexposed control children (UC). There was a modestly elevated incidence rate of epilepsy at one year of life in EC, in the absence of any other apparent neurological manifestations. This rate represents the first time point in a longitudinal series. Continued surveillance of this cohort will determine whether epilepsy rates increase with continuing brain maturation in EC, relative to rates in the parallel group of UC.

World Health Organization recommendations for screening, assessing, and managing neonates and infants in areas of ZIKV transmission include a full neurological examination, neuroimaging, and continued monitoring for seizures throughout infancy when microcephaly is present [24]. However, guidelines for continued surveillance of normocephalic children are less specified. It is recommended that normocephalic children with suspected, probable, or confirmed ZIKV exposure receive neurodevelopmental assessments at a minimum of 3, 9, and 24 months of age [24]. Our results support the inclusion of a brief pediatric epilepsy screening questionnaire [20] in these follow-up evaluations to probe whether the child is having seizures and if so, to specify features of the episodes that can help distinguish focal from generalized epilepsy syndromes.

Prior case series that have investigated epilepsy profiles in children with Congenital Zika Syndrome (CZS) have only included children with microcephaly and/or craniofacial disproportions, arthrogryposis, and motor abnormalities [1–5]. The prevalence of epilepsy in these case series was high, ranging from 48–96%, and the most common seizure types were infantile spasms and focal seizures [4,5]. Seizure onset was typically in the first few months of life and the most predominant electroencephalographic (EEG) findings were abnormal background rhythms, hypsarrhythmia, and focal or multifocal epileptiform discharges [4,5]. Good seizure control was achieved in approximately 65% of children with CZS and microcephaly, more frequently with polytherapy [4].

Our study findings suggest that the epilepsy profile in normocephalic EC may have similar features to more severe CZS-associated epilepsy but also important differences. In our positive epilepsy cases, questionnaire responses indicated focal seizure features. There was no evidence for generalized epileptiform discharges or hypsarrhythmia on interictal vEEGs, which increased confidence in diagnosis of a focal epilepsy syndrome. Although the number of cases was small, our results cautiously raise the possibility that epilepsy in normocephalic EC might be associated with focal brain malformations that are on the milder end of the CZS spectrum [12,39]; however, high-resolution neuroimaging is needed for confirmation.

Clinical evidence for ZIKV-associated seizures in the context of more subtle congenital brain malformations is limited in humans; however, there is strong evidence in mice, with a high incidence of seizures and focal spiking activity in normocephalic ZIKV-infected mice that had neuropathological findings of calcifications and cytoarchitectural disruption [40]. Although we observed only a modest elevation of epilepsy rates in EC during their first year of life, it is possible that ZIKV-associated seizures can emerge at a later stage of development in humans. Seizures associated with focal cortical dysplasia typically emerge in early childhood [41]; however, onset can range from 3 to 35 years [42,43]. ZIKV mouse models also show increased susceptibility to chemically-induced seizures, raising the possibility that a “second hit” (e.g., illness, stress, or injury) may be needed to induce seizure onset in EC.

Continued surveillance is needed to better characterize lifetime epilepsy risk in EC, identify potential “second hits,” and characterize the developmental trajectory of seizure expression. It is unclear whether seizures in EC will worsen over time, resist medication, or potentially resolve. ZIKV mouse models show resolution of “infantile” seizures in the majority of infected normocephalic mice [40], which suggests that EC-associated epilepsy may show a unique developmental pattern. Continued surveillance of our cohort provides a valuable opportunity to address these unanswered questions, with the current results serving as a baseline from which to compare future findings.

Our study also demonstrates that large-scale epilepsy surveillance is feasible in resource-limited LMICs in the event of future ZIKV outbreaks. In several ZIKV-endemic countries throughout the Caribbean, there are no neurologists, EEG, or MRI services [14]. We trained non-specialist local research staff to administer the pediatric epilepsy screening questionnaire and vEEG, which allowed for broader surveillance and follow-up with specialists in the event of positive screens. The screening questionnaire was low cost (printing costs only), quick to administer (less than five minutes), and easily understood by caregivers after local adaptation of the language (i.e., “fits” for seizures). Importantly, one of the children diagnosed with epilepsy was identified by our screening procedures, which led to an evaluation, treatment, and good seizure control. This illustrates that epilepsy surveillance can reduce the time to treatment, potentially improving outcomes in at-risk children.

The *micro*EEG system that we utilized was easy to apply and well tolerated by the 12- to 30-month-old children that we screened. We did not use any form of sedation but were able to keep the majority of children from moving with behavioral methods, such as modeling of procedures (i.e., applying the EEG to a doll with the child in advance), encouraging the child to sit on their caregiver’s lap, and reading or watching videos while recording. Ultimately, 94% of the vEEGs were considered interpretable, which demonstrates the feasibility of obtaining high quality vEEG data in remote, high-noise, resource-limited settings. There were no differences in the rates of abnormal vEEG findings between the EC and UC groups. This suggests that vEEG may not be necessary as a screening tool in EC without any apparent neurologic manifestations. However, results from our study support the use of vEEG in positive epilepsy screening cases to assist in epilepsy diagnosis.

There are several limitations in our study that warrant caution when generalizing findings to the larger population of EC. First, our study sample was small, and the incidence of epilepsy was rare. Reasons for the small sample size include challenges in determining ZIKV-exposure status in more than a third of our sample whose mothers were enrolled in the postnatal period, as well as challenges in getting families to return for follow-up epilepsy screening procedures. Results should be considered preliminary; they support the use of our epilepsy surveillance protocol in a large-scale follow-up study of EC from different regions of the Caribbean and Latin America to determine the prevalence of epilepsy, particularly as EC continue to mature. Second, by including all pregnant mothers, including those who were asymptomatic, we were unable to identify the trimester of infection in the majority of the sample. Although this approach increased the uncertainty in timing of infection, it allowed for a broader sample of symptomatic and asymptomatic ZIKV-infected women and avoids the ascertainment bias of only including symptomatic mothers and/or children. Third, cross-reactivity within the flaviviral family is a concern when ZIKV infection status is determined by serological assay; however, the pGOLD platform that we utilized is specifically designed to minimize cross-reactivity in the differential diagnosis of ZIKV and DENV with high sensitivity and specificity [22]. Although results suggest that 20% of the mothers with positive laboratory results for ZIKV may have also been infected with DENV during pregnancy, this is unlikely to have impacted our findings as one of the children with epilepsy was born to a mother with evidence of more remote (i.e., more than 6 months) DENV infection; whereas, the other child was born to a mother with evidence of more proximal (i.e., less than 6 months) DENV infection. Fourth, a larger number of EC completed vEEG procedures relative to UC, which may have been due to a disinclination for mothers of UC to consent to the lengthier vEEG procedures. However, this had little impact on our main study findings, as epilepsy classification primarily relied on the presence of clinical seizures, not vEEG findings. Finally, a strength of our study is that head circumference measurements were obtained at birth and between 12 and 30 months, assuring that none of the EC developed late onset microcephaly, which has been previously described in ZIKV-exposed children who were normocephalic at birth [44].

## Conclusion

Epilepsy surveillance procedures should be included in the neurodevelopmental assessment of children with suspected, probable, or confirmed *in utero* ZIKV exposure during their first two years of life, even if no neurologic manifestations are present at birth. A pediatric epilepsy screening questionnaire is low cost and high yield and can be administered by non-specialists in resource-limited ZIKV-endemic regions in the event of future ZIKV outbreaks.
